# The Autism–Tics, ADHD and other Comorbidities inventory (A-TAC): previous and predictive validity

**DOI:** 10.1186/s12888-017-1563-0

**Published:** 2017-12-16

**Authors:** Caroline Mårland, Paul Lichtenstein, Alessio Degl’Innocenti, Tomas Larson, Maria Råstam, Henrik Anckarsäter, Christopher Gillberg, Thomas Nilsson, Sebastian Lundström

**Affiliations:** 1 Centre for Ethics Law and Mental Health (CELAM) Institute of Neuroscience and Physiology, University of Gothenburg, SU Rättspsykiatri, Rågårdsvägen 5, Enhet CELAM Hus 1, SE-424 57 Gunnilse Gothenburg, Sweden; 2 Department of Forensic Psychiatry, Sahlgrenska University Hospital, SU Rättspsykiatri, Rågårdsvägen 5, SE-424 57 Gunnilse Gothenburg, Sweden; 3 Department of Medical Epidemiology and Biostatistics, Karolinska Institutet, Box 281, SE-171 77 Stockholm, Sweden; 4 Department of Clinical Sciences, Lund University, Box 117, SE-221 00 Lund, Sweden; 5 Gillberg Neuropsychiatry Centre Institute of Neuroscience and Physiology, University of Gothenburg, Kungsgatan 12, SE-411 19 Göteborg, Sweden

**Keywords:** Autism, Neurodevelopmental disorders, ADHD, Screening

## Abstract

**Background:**

Reliable and easy to administer screening instruments focusing on neurodevelopmental disorders and associated conditions are scarce. The Autism–Tics, AD/HD and other Comorbidities inventory (A-TAC) has previously been validated and reporting good– excellent validity for several disorders. This article aims to expand these findings by including more conditions in a substantially larger sample augmented with the Swedish National Patient Register (NPR).

**Methods:**

Since 2004 parents of all 9-year-old Swedish twins have been invited to participate in a telephone interview in the Child and Adolescent Twin Study in Sweden, CATSS. The CATSS is linked to the NPR which includes data from in- and outpatient care. Data on neurodevelopmental disorders (A-TAC) collected in CATSS were compared with diagnoses from the NPR. We investigated diagnoses that had been made both before (previous validity) and after (predictive validity) the interview.

**Results:**

Sensitivity and specificity of A-TAC scores for predicting earlier or later clinical diagnoses were mostly good–excellent, with values of the area under the curve for a clinical diagnosis of autism spectrum disorder (ASD) of .98, attention deficit hyperactivity disorder (ADHD) .93, learning disorder (LD) .92, and oppositional defiant disorder (ODD) .99, with small differences in terms of previous and predictive analyses. A-TAC provided little validity for eating disorders.

**Conclusion:**

The result support previous claims: A-TAC is a broad screening instrument with a particular strength in assessing ASD, ADHD, LD, and ODD at ages 9 and 12, and also provides phenotypic information about other child psychiatric disorders.

**Electronic supplementary material:**

The online version of this article (10.1186/s12888-017-1563-0) contains supplementary material, which is available to authorized users.

## Background

Childhood-onset neuropsychiatric disorders, commonly lumped together under the concept of neurodevelopmental disorders (NDDs), affect at least 7–10% of all children [1]. They have traditionally been considered as distinct and separate from each other, each with a course of its own. However, the term Early Symptomatic Syndromes Eliciting Neurodevelopmental Clinical Examinations (ESSENCE) has been suggested in order to emphasize the clinical reality of children as manifestations of one or more major developmental symptoms before the age of 5 often implies problems in the same or an overlapping area later in life [1]. Today it is acknowledged that NDDs share symptomatology [1, 2] follow a waxing and waning course [3], and can be unstable within a diagnostic category over time [4, 5]. In addition, NDDs share predisposing environmental and genetic factors with each other [6], and are dimensionally distributed in the population [7]. Taken together, validated screening instruments that dimensionally assess the symptomatology of several NDDs simultaneously are therefore required as a complement to the diagnostic interview in clinical practice, but also in large-scale epidemiological studies where diagnostic assessments are rarely feasible.

The Autism–Tics, AD/HD and other Comorbidities inventory (A-TAC) was developed by three of the authors (CG, MR, and HA) at the Department of Child and Adolescent Psychiatry, forerunner of the Gillberg Neuropsychiatry Centre, at the University of Gothenburg. The A-TAC is a broad-band screening instrument that encompasses autism spectrum disorder (ASD), attention deficit hyperactivity disorder (ADHD), tic disorders (TD), developmental coordination disorder (DCD), and learning disorder (LD). Furthermore, the A-TAC also covers possible overlapping childhood neuropsychiatric and/or psychiatric disorders such as obsessive-compulsive disorder (OCD), oppositional defiant disorder (ODD), conduct disorder (CD), and eating disorders (ED). All disorders targeted in the A-TAC have been subjected to validation studies on children and adolescents: Three cross-sectional validation studies [8–10] and one longitudinal [11]. Briefly, cross-sectionally the A-TAC shows excellent inter-rater reliability and good test-retest measures [8, 10]. The ASD and ADHD modules have shown excellent screening properties in a clinical sample of children when compared to diagnoses assigned during an investigation at a child neuropsychiatric clinic [8]. These results were later replicated by Larson et al. in a community based sample of children, for ASD and ADHD the “low” cut-off value for screening purposes reported a high sensitivity (above 0.95) and the clinical proxy reported a high specificity (0.95) [9]. Cut-off scores for DCD, LD, and TD have also been established in clinical as well as community samples [8, 9]. A longitudinal three-year follow-up study of 452 individuals showed that the A-TAC could be used as a predictive assessment tool: the predictive validity was good to excellent for ASD, ADHD, LDs, and TDs [11]. The A-TAC showed convergent validity with the Child Behavior Checklist in scales with related content, but also provided a more specific assessment of ASDs, DCD, LD and tic disorders [12]. In addition, the ASD module has been subjected to an independent Spanish validation [13] where the psychometric properties were reported to be excellent.

Even though the A-TAC can be considered to be well-validated there are still some issues that need to be addressed: (a) The number of cases in the longitudinal validation study were limited as some NDDs were rare, for instance the clinically assessed ASD-group consisted of 20 individuals only. Conditions like CD and ODD are overrepresented among non-responders, and were not sufficiently represented in the validation studies. (b) The onset of EDs are generally later than the assessed ages [14], leading to an underrepresentation of this type of disorders, why it has been difficult to establish the predictive validity for these disorders. (c) Those who participate in the clinical validation studies might differ in their degree of symptomatology, dysfunction, and suffering, compared to those who do not participate. Therefore previous validation studies of the A-TAC might not satisfactorily investigate the instruments ecological validity.

A-TAC has been used in an ongoing nation-wide twin study named the Child and Adolescent Twin Study in Sweden (CATSS) that has been linked to the National Patient Register (NPR). This makes it possible to, among other things, study the validity of the A-TAC in a population-based sample, and thus get even more reliable results for rare conditions, and in conditions where non-responding is elevated. A distinction between previous and predictive validity was made. This distinction was motivated by the fact that CATSS has been ongoing for more than 10 years. During this time differences in diagnostic practice, professional and public awareness and referral patterns have most likely arisen. For instance, the high cut-off for ASD has remained stable for 10 years in CATSS while the officially recorded prevalence in NPR has increased substantially [15]. Furthermore, there is substantial evidence that secular changes in practice are related to diagnostic substitution. Shattuck reported a decreasing prevalence of learning disabilities and intellectual disability in USA from 1994 to 2003 with an equivalent increasing prevalence of ASD [16].

The aim of this study was to investigate the previous and predictive validity of the A-TAC in a nation-wide sample of twins where register-based diagnoses were available.

## Method

### The Child and Adolescent Twin Study in Sweden (CATSS)

The CATSS is an ongoing longitudinal twin study designed to assess somatic and mental health disorders during childhood. The study has been described in detail in an overview-article [17]. Briefly, since July 2004, parents of all Swedish 9-year-old twins (born 1st July 1995 and onwards) were identified through the Swedish Twin Registry and asked to participate in a telephone interview. During the first three years of the CATSS, 12-year-old twins (born July 1992–June 1995) were also included. The rationale for choosing these age groups were that most major childhood mental disorders have emerged by then, while the normal problems of puberty have not. The CATSS has a response rate of 75% with small differences between responders and non-responders with regards to psychopharmacological treatment and socioeconomic status. In 87,5% of the interviews the informant was the mother; 12,2% the father, and for the remaining 0,3% another member of the family [17].

### The Swedish National Patient Register (NPR)

All individuals who are born in Sweden or have received a citizenship are given a personal identification number, which is recorded in all contacts with health care, social- and administrative services. The CATSS data was merged with the NPR via the personal identification number. The NPR includes information about all ascribed diagnoses in psychiatric inpatient care since 1987 [18]. Since 2001 the register also includes data from outpatient consultations. The NPR consists of best-estimate specialist diagnoses assigned according to the International Classification of Diseases ninth (ICD-9) and tenth (ICD-10) revision [19, 20]. The validity of data in the NPR is continuously under assessment: A validation study, which included NPR diagnoses, reported that 96% (confidence interval 92.0%–98.4%) of the listed ASD diagnoses could be confirmed through medical records [21]. Similarly the validity of OCD and TD is reported to be high [22]. Data from the NPR was available up until 31st of December 2012.

The population for which data from the NPR was extracted consisted of 25,828 twins (49% ♀), 19,322 9-year-olds and 6,506 12-year-olds whose parents had responded to the A-TAC. The clinicians who ascribed the diagnoses in the NPR did not have any knowledge about the result from the A-TAC interview.

### Measures

#### The Autism–Tics, AD/HD and other Comorbidities inventory (A-TAC)

The A-TAC is a collateral interview fully structured to enable laymen to preform it over the telephone. It consists of 96 items based on the diagnostic criteria in the Diagnostic and Statistical Manual of Mental Disorders, fourth edition (DSM-IV) [23], clinical experience and relevant aspects or questions included in available screening instruments in the field of NDD, such as the Asperger Syndrome Screening Questionnaire [24], the Asperger Syndrome Diagnostic Interview [25] and the Five to Fifteen questionnaire [26].

The items are divided into 20 theoretically defined modules: Motor Control; Perception; Concentration & Attention; Impulsiveness & Activity; Learning; Planning & Organizing; Memory; Language; Social Interaction; Flexibility; Tics; Compulsions; Feeding; Separations; Opposition; Conduct; Anxiety; Mood; Concept of Reality; and Miscellaneous (i.e. clinically specific problems that are not covered by the other modules such as sleep, food fads, severe overweight, bodily functions, and substance abuse). Each module is assessed without taking diagnostic exclusion or hierarchies into account. The items are assessed in a “whole-life” frame and are coded on a dimensional scale where each item has three response categories:” No” scored as 0,” Yes, to some extent” scored as 0.5, and” Yes” scored as 1. The A-TAC has an average administration time of ≈ 27.5 min and is frequently used in clinical practice [10]. It is an open access instrument that is available in Swedish and English and can be downloaded from the Gillberg Neuropsychiatry Centre website [27] and is also available as an appendix in Larson et al. [9].

### Definition of disorders

#### A-TAC

Each A-TAC module corresponds to a diagnostic domain, except the ASD and ADHD domains, which include several modules. In Table 1 the A-TAC modules, total number of items, previously reported internal consistency as well as sensitivity and specificity for each established cut-off value are presented. For ASD, ADHD, LD, and DCD, two cut-off values have been created: a “low” cut-off with high sensitivity for broad screening purposes and a “high” cut-off with a high specificity to use as a clinical proxy in research settings [9]. For ODD and CD clinical proxies has been created [28]. No validation has been conducted for the OCD and ED-modules.

**Table 1:** The A-TAC Modules: Previously Reported Internal Consistency and Sensitivity/Specificity for each Established Cut-off Value

Diagnostic domain	A-TAC module	Items	Cronbach’s α	Cut-off	sens/spec
ASD	Language, Social Interaction	17	.86^b^	4.5 (low)	0.91/0.80^c^
	and Flexibility			8.5 (high)	0.61/0.91^c^
	*- Language*	*6*	*.66* ^*b*^		
	*- Social Interaction*	*6*	*.77* ^*b*^		
	*- Flexibility*	*5*	*.70* ^*b*^		
ADHD	Concentration & Attention and	19	.92^b^	6 (low)	0.91/0.73
	Impulsiveness & Activity			12.5 (high)	0.56/0.93^c^
	*- Concentration & Attention*	*9*	*.90* ^*b*^		
	*- Impulsiveness & Activity*	*10*	*.87* ^*b*^		
LD	Learning	3		1 (low)	0.92/0.60^c^
				3 (high)	0.41/0.93^c^
DCD	Motor control	1		0.5 (low)	0.63/0.68^c^
				1 (high)	0.32/0.87^c^
TD	Tics	3	.57^b^	1.5	0.875/0.86^c^
ODD	Opposition	5	.75^b^	3	0.51/0.96^d^
CD	Conduct	5	.61^b^	2	0.55/0.98^d^
OCD^a^	Compulsions	2		1	
ED^a^	Feeding	2		1	

^a^No previous established cut-off value

^b^Anckarsäter et al., 2011

^c^Larson et al., 2010

^d^Kerekes et al., 2014

#### NPR

From the NPR, diagnostic data were obtained by searching for ICD-9 and ICD-10 codes to the corresponding conditions. If only one diagnostic code is presented, it includes all types subsumed under that code. ASD (ICD-9: 299.0, 299.8, 299.9, ICD-10: F84.0, F84.1, F84.5, F84.9), ADHD (ICD-9: 314, ICD-10: F90), LD (ICD-9: 317–319, ICD-10: F70-F79), DCD (ICD-9: 315.4, ICD-10: F82), TD (ICD-9: 307.2, ICD-10: F95), ODD (ICD-9: 313.8, ICD-10: F91.3), CD (ICD-9: 312, ICD-10: F91, excluding F91.3), OCD (ICD-9: 300.3, ICD-10: F42), ED (ICD-9: 307.1, 307.50, 307.51, ICD-10: F50).

### Statistical analysis

The sample was divided into two different groups based on age when the first diagnosis was listed in the NPR: (1) before (previous) or (2) after (predictive) the A-TAC interview. The previous-group also included twins who had the first diagnosis registered the same year as the A-TAC interview. The analyses were also conducted in a collapsed fashion, including the whole sample. Since the CATSS included 12 year-old twins the first three years, calculations based on the A-TAC were also stratified by age.

Receiver operating characteristics (ROC) was used in order to illustrate the area under the curve (AUC) for the different scale steps. The AUC is a measure of how good the instrument can discriminate between false positives and true positives on a binary classifier (diagnosis) for all possible values on a continuous scale and provides information about the sensitivity and specificity for each scale step. An AUC equal to 0.50 indicate random prediction, 0.60–0.70 poor validity, 0.70–0.80 is fair, 0.80–0.90 is good and an AUC > 0.90 signals excellent validity [29]. The scales from A-TAC were used as independent predictors and the clinical diagnoses from the NPR were used as dependent variables.

Each cut-off value was examined by measures of sensitivity (sens), specificity (spec), positive predictive value (PPV) and negative predictive value (NPV). Sensitivity is defined as the proportion of subjects with a true disorder who are identified as screen-positives while specificity defines the proportion of subjects without a disorder who are identified as screen-negatives. The predictive values, PPV and NPV, are dependent on the prevalence of a disorder. PPV describes the probability that a positive prediction is correct and NPV the probability that a negative prediction is correct [30, 31].

In order to interpret the results irrespective of base rates and the “false positive paradox” (i.e., when the false-positive rate is higher than the actual prevalence of the studied condition) we included the diagnostic odds ratio (DOR) which is defined as the ratio of odds of a screen-positive result for a subject with a true disorder relative to the odds of a screen-positive result for a non-afflicted subject. The DOR value ranges from zero to infinity and higher DOR values suggest a better discriminatory test performance, and is such a helpful tool when deciding how accurate a diagnostic test is and how it compares to other tests [30, 32].

All statistical analyses were performed in the SPSS software package, version 22.0.

### Ethical considerations

Informed consent was obtained from all parents. The data collection in the CATSS, and the linkage to the NPR has ethical approval from the Karolinska Institute ethical review board (Dnr 02–289 and 2010/507–31/1).

## Results

The prevalence of the diagnoses according to the NPR and the number of subjects with an A-TAC score equal to or higher than each cut-off value are presented in Table 2. The previous and predictive validities of the A-TAC are reported in Table 3. The previous validity for ASD was excellent (AUC = 0.98). The screening cut-off showed a high sensitivity (0.85) and the clinical proxy had a high specificity (0.99). The NPV was high (0.999 and 0.997 respectively), the clinical proxy had a higher PPV (0.22) while the screening cut-off had a higher DOR (166). The LD module reported an excellent predictive validity (AUC = 0.92). The screening cut-off showed a higher sensitivity (0.89) than specificity (0.85) and the clinical proxy had a high specificity (0.99) but a considerably lower sensitivity (0.40). The NPV was high (0.999 and 0.998 respectively) while the PPV was low for both cut-off values (0.02 and 0.1), the screening cut-off showed the highest DOR value (48).

**Table 2:** Subjects With a Listed Disorder in the NPR (%) and Screen-positives in A-TAC (%)

	Disorder in the NPR			A-TAC	
Disorder	Before	After	Total	Cut-off	Screen-positive
ASD	127 (0.5)	171 (0.7)	298 (1.2)	4.5	924 (3.6)
				8.5	266 (1.0)
ADHD	231 (0.9)	492 (1.9)	723 (2.8)	6	2707 (10.5)
				12.5	524 (2.0)
LD	145 (0.6)	104 (0.4)	249 (1.0)	1	3961 (15.3)
				3	428 (1.7)
DCD	70 (0.3)	18 (0.1)	88 (0.3)	0.5	2050 (7.9)
				1	469 (1.8)
TD	41 (0.2)	47 (0.2)	88 (0.3)	1.5	847 (3.3)
ODD	8 (0.0)	20 (0.1)	28 (0.1)	3	795 (3.1)
CD	27 (0.1)	50 (0.2)	77 (0.3)	2	278 (1.1)
OCD^a^	9 (0.0)	61 (0.2)	70 (0.3)	1	479 (1.9)
ED^a^	32 (0.1)	127 (0.5)	159 (0.6)	1	1371 (5.4)

*N* = 25,828

Disorder in NPR: Ascribed diagnoses in NPR before and after the telephone interview (CATSS) and in total

A-TAC: Number of screen-positive subjects with an A-TAC score equal to or higher than each cut-off value

Please see the supplementary material for numbers of true positive, false positive, true negative and false negative for all disorders (Additional file 1), and the sensitivity and specificity for each point on ASD and ADHD scale (Additional file 2)

^a^No previous established cut-off value

**Table 3:** Previous and Predictive Validity of A-TAC

Disorder	Cut-off	Previous					Predictive					Total				
		AUC	sens/spec	PPV	NPV^b^	DOR	AUC	sens/spec	PPV	NPV^b^	DOR	AUC	sens/spec	PPV	NPV^b^	DOR
ASD	4.5 (low)	0.98	0.85/0.97	0.11	0.999	166	0.81	0.42/0.97	0.08	0.996	21	0.89	0.60/0.97	0.19	0.995	49
	8.5 (high)		0.48/0.99	0.22	0.997	113		0.17/0.99	0.11	0.995	22		0.30/0.99	0.33	0.992	62
ADHD	6 (low)	0.93	0.79/0.90	0.07	0.998	34	0.82	0.56/0.90	0.10	0.991	12	0.86	0.64/0.91	0.17	0.989	18
	12.5 (high)		0.46/0.98	0.2	0.995	50		0.19/0.98	0.18	0.984	14		0.28/0.99	0.38	0.979	29
LD	1 (low)	0.87	0.83/0.85	0.03	0.999	27	0.92	0.89/0.85	0.02	0.999	48	0.89	0.86/0.85	0.05	0.998	34
	3 (high)		0.37/0.99	0.13	0.996	40		0.40/0.99	0.1	0.998	44		0.39/0.99	0.22	0.994	48
DCD	0.5 (low)	0.82	0.70/0.92	0.02	0.999	27	0.57	0.22/0.92	0	0.999	3	0.77	0.60/0.92	0.03	0.999	18
	1 (high)		0.38/0.98	0.06	0.998	35		0.11/0.98	0	0.999	7		0.32/0.98	0.06	0.998	27
TD	1.5	0.86	0.59/0.97	0.03	0.999	43	0.80	0.47/0.97	0.03	0.999	27	0.83	0.52/0.97	0.05	0.998	34
ODD	3	0.99	0.88/0.97	0.01	1.0	222	0.80	0.45/0.97	0.01	1.0	26	0.85	0.57/0.97	0.02	1.0	43
CD	2	0.90	0.52/0.99	0.05	0.999	104	0.70	0.20/0.99	0.04	0.998	24	0.77	0.31/0.99	0.09	0.998	45
OCD^a^	1	0.88	0.67/0.98	0.01	1.0	107	0.65	0.25/0.98	0.03	0.998	18	0.68	0.30/0.98	0.04	0.998	24
ED^a^	1	0.72	0.38/0.95	0.01	0.999	11	0.52	0.10/0.95	0.01	0.995	2	0.56	0.16/0.95	0.02	0.995	3

Previous and predictive validity: area under the curve (AUC), sensitivity/specificity, positive (PPV) and negative predictive value (NPV), and diagnostic odds ratio (DOR) for each cut-off value in the A-TAC

^a^No previous established cut-off value

^b^For clarity reasons, NPV is reported with three decimals

The results for the age-specific groups, i.e. 9- respective 12-year-olds, are available as supplementary material (Additional file 3). These results showed a similar pattern as the collapsed group with the only exception for the OCD module for the 12-year olds which displayed an excellent predictive validity (AUC = 0.99) with an excellent sensitivity (1.0) and specificity (0.98).

## Discussion

The purpose of this study was to examine the previous and predictive validity of the A-TAC inventory in a nation-wide sample of twins. The findings in this study support previous claims: A-TAC has a particular strength in assessing ASD, ADHD, LD, and ODD at ages 9 and 12, and also provides phenotypic information about other child psychiatric disorders. A-TAC has the ability to identify subjects without NPD, which is valuable in epidemiological research and in case-control studies. In clinical practice, the “low” cut-off values should be used in order to get an initial assessment that makes it possible to directly focus on the child’s most predominant symptoms and behavioral problems.

The results suggest that the A-TAC has good validity for previously made diagnoses while its predictive validity (with the exception of LDs) is not as good. This, possibly, reflects the fact that parents of already diagnosed children had knowledge about the disorders in question and thus were better able to recognize the symptoms that were asked about during the telephone interview. Future studies using the A-TAC should take this discrepancy into account. Nevertheless, given the difference in AUCs between the “previous” and “predictive” groups it cannot be ruled out that the age of a given diagnosis indicate a difference in the studied phenotype. ASD and ADHD diagnosed in adolescence may differ on a number of features compared to traditional view, especially considering the rising prevalences in Sweden [33]. That is, the impact of the disorder on psychological abilities and adaptive behavior might differ in severity and extent so that treatment seeking is postponed until several years after the age of nine. Information derived from the A-TAC for other age-groups than 9 and 12 years may also be less reliable and adhere to different cutoffs than those proposed here. Particular attention is warranted in the case of individuals aged >12 when features “typical” of adolescence and puberty, rather than neurodevelopmental disorders, may influence the rating. Furthermore, for teenagers in clinical settings the low sensitivity of the ASD low cut-off (.42) argues against its use as an instrument to select out those who need further assessments. Future research should include the exploration of age- and gender specific cutoffs so that psychometric properties of the A-TAC would be available, and also more specific, for different groups. For instance, for OCD the mean age of onset has been reported to be 11.1 years [34], which becomes evident as excellent previous validity for individuals aged 12 was reported, but not for those assessed at age 9.

A-TACs dimensionality and together with its broad encompassing of several neurodevelopmental disorders can aid in research as well as in clinical settings. When considering assessment and treatment of neurodevelopmental disorders, the co-existence of ‘other’ disorders is essential. For instance, ASD in itself may not confer an increased risk for negative outcomes [35], but coexisting disorders, including ADHD, learning disabilities, language disorder, tic disorders and other disorders (and combinations of these) may be the real mediators when it comes to negative outcomes [35–37]. Furthermore, in addition to studying the effects of categorical diagnoses, there is a need to include continuous measures of NDPs [38], the rationale being that neurodevelopmental disorders tend to oscillate above and below the diagnostic threshold. It is also unclear how the presence of subthreshold traits from one category influences the prognosis of individuals with ‘another’ disorder should be considered.

The present study has several strengths, including a large population-based sample, a long follow-up time (i.e. 20 years for those born 1992), a high response rate and linkage to a national patient register of good quality. However, there are also some limitations to this study that warrant discussion. The main limitation consists of the diagnostic categories with a low number of individuals (≤ 1%), i.e. DCD, TD, ODD, CD, OCD and ED, which may yield uncertainties about the observed result. The reason for the low prevalence of these disorders in the NPR is unknown, but clinicians may prefer to assign a principal diagnosis without any secondary diagnoses. For instance, clinicians might “expand” the ADHD symptomatology to include ODD and CD symptoms or the ASD symptomatology to include DCD. Furthermore, it has been argued that twins differ from singletons and that twins may not be representative of the general population in terms of neurodevelopmental disorders. It has also been suggested that twinning could increase the risk of developing ASD [39, 40]. However, this notion has not been confirmed in large population-based studies [15, 41]. Also, studies of general psychiatric outcomes in twins, such as psychotic and affective disorders, have found no or small differences between twins and singletons [42–44].

## Conclusions

The A-TAC showed good to excellent validity for a majority of the studied diagnoses, both in previous and predictive data, and is a suitable screening tool for a wide range of NDDs at ages 9 and 12.

## Additional files

Additional file 1: Cross tables. Include cross tables that present the numbers of true positive, false positive, true negative and false negative for all disorders in the total group. (DOCX 23 kb)

Additional file 2: ASD and ADHD sensitivity and specificity. The tables present the estimates from the ROC-curves for ASD and ADHD. Sensitivity and specificity values are presented for each possible A-TAC score. (DOCX 23 kb)

Additional file 3: CATSS data stratified by age. **Table S1.** and **Table S2.** include data from the 12- year-olds, born 1st of July 1992 to 30th of June 1995. **Table S3.** and **Table S4.** include data from the 9-year-olds, born 1st of July 1995 and onwards. (DOCX 31 kb)

## Abbreviations

ADHD: Attention deficit hyperactivity disorder
ASD: Autism spectrum disorder
A-TAC: Autism–Tics, AD/HD and other Comorbidities inventory
AUC: Area under the curve
CATSS: Child and Adolescent Twin Study in Sweden
CD: Conduct disorder
DCD: Developmental coordination disorder
DOR: Diagnostic odds ratio
DSM: Diagnostic and Statistical Manual of Mental Disorders
ED: Eating disorders
ESSENCE: Early symptomatic syndromes eliciting neurodevelopmental clinical examinations
ICD: International Classification of Diseases
LD: Learning disorder
NDD: Neurodevelopmental disorders
NPR: National patient register
NPV: Negative predictive value
OCD: Obsessive-compulsive disorder
ODD: Oppositional defiant disorder
PPV: Positive predictive value
ROC: Receiver operating characteristics
Sens: Sensitivity
Spec: Specificity
TD: Tic disorders
