# Supplementary material for: The Autism–Tics, ADHD and other Comorbidities inventory (A-TAC): previous and predictive validity
Source: BMC Psychiatry. 2017 Dec 16;17:403. doi: 10.1186/s12888-017-1563-0 (PMC5732476; doi:10.1186/s12888-017-1563-0)
Supplement: Supplementary file 1 — Cross tables. Include cross tables that present the numbers of true positive, false positive, true negative and false negative for all disorders in the total group. (DOCX 23 kb) [file 12888_2017_1563_MOESM1_ESM.docx]

**Cross tables**

The following tables present the numbers of true positive, false positive, true negative and false negative for all disorders. The rows in the tables present the number of screen-positive or screen-negative subjects in A-TAC for each cut-off value. The columns present the number of subjects with or without a registered disorder in NPR.

**Disorder: ASD**

| **Cut-off: 4.5*** | ASD | No ASD |  | **Cut-off: 8.5*** | ASD | No ASD |  |
| --- | --- | --- | --- | --- | --- | --- | --- |
| Screen-positive | 174 | 750 |  | Screen-positive | 88 | 178 |  |
| Screen-negative | 117 | 24750 |  | Screen-negative | 203 | 25322 |  |
| *Missing: 37 | | |  | *Missing: 37 | | | |

**Disorder: ADHD**

| **Cut-off: 6*** | ADHD | No ADHD |  | **Cut-off: 12.5*** | ADHD | No ADHD |
| --- | --- | --- | --- | --- | --- | --- |
| Screen-positive | 456 | 2251 |  | Screen-positive | 199 | 325 |
| Screen-negative | 261 | 22794 |  | Screen-negative | 518 | 25045 |
| *Missing: 66 | | |  | *Missing: 66 | | |

**Disorder: LD**

| **Cut-off: 1*** | LD | No LD |  | **Cut-off: 3*** | LD | No LD |
| --- | --- | --- | --- | --- | --- | --- |
| Screen-positive | 213 | 3748 |  | Screen-positive | 96 | 332 |
| Screen-negative | 36 | 21831 |  | Screen-negative | 153 | 25247 |
| *Missing: 0 | | |  | *Missing: 0 | | |

**Disorder: DCD**

| **Cut-off: 0.5*** | DCD | No DCD |  | **Cut-off: 1*** | DCD | No DCD |
| --- | --- | --- | --- | --- | --- | --- |
| Screen-positive | 52 | 1998 |  | Screen-positive | 28 | 441 |
| Screen-negative | 35 | 23741 |  | Screen-negative | 59 | 25298 |
| *Missing: 2 | | |  | *Missing: 2 | | |

**Disorder: TD**

| **Cut-off: 1.5*** | TD | No TD |
| --- | --- | --- |
| Screen-positive | 46 | 801 |
| Screen-negative | 42 | 24915 |
| *Missing: 22 | | |

**Disorder: ODD**

| **Cut-off: 3*** | ODD | No ODD |
| --- | --- | --- |
| Screen-positive | 16 | 779 |
| Screen-negative | 12 | 24981 |
| *Missing: 40 | | |

**Disorder: CD**

| **Cut-off: 2*** | CD | No CD |
| --- | --- | --- |
| Screen-positive | 24 | 254 |
| Screen-negative | 53 | 25467 |
| *Missing: 30 | | |

**Disorder: OCD**

| **Cut-off: 1*** | OCD | No OCD |
| --- | --- | --- |
| Screen-positive | 21 | 458 |
| Screen-negative | 49 | 25298 |
| *Missing: 2 | | |

**Disorder: ED**

| **Cut-off: 1*** | OCD | No OCD |
| --- | --- | --- |
| Screen-positive | 25 | 1346 |
| Screen-negative | 134 | 24318 |
| *Missing: 5 | | |
