# Supplementary material for: The Autism–Tics, ADHD and other Comorbidities inventory (A-TAC): previous and predictive validity
Source: BMC Psychiatry. 2017 Dec 16;17:403. doi: 10.1186/s12888-017-1563-0 (PMC5732476; doi:10.1186/s12888-017-1563-0)
Supplement: Supplementary file 2 — ASD and ADHD sensitivity and specificity. The tables present the estimates from the ROC-curves for ASD and ADHD. Sensitivity and specificity values are presented for each possible A-TAC score. (DOCX 23 kb) [file 12888_2017_1563_MOESM2_ESM.docx]

**ASD and ADHD: sensitivity and specificity**

The following tables present the estimates from the ROC-curves for ASD and ADHD. Sensitivity and specificity values are presented for each possible A-TAC score.

**Disorder: ASD (Total)**

| **A-TAC score** | **Sensitivity** | **Specificity** |
| --- | --- | --- |
| 0 | 1.0 | .0 |
| .5 | .911 | .531 |
| 1 | .873 | .708 |
| 1.5 | .821 | .817 |
| 2 | .801 | .874 |
| 2.5 | .763 | .913 |
| 3 | .708 | .934 |
| 3.5 | .667 | .951 |
| 4 | .625 | .963 |
| 4.5 | .598 | .971 |
| 5 | .567 | .976 |
| 5.5 | .533 | .98 |
| 6 | .515 | .984 |
| 6.5 | .481 | .986 |
| 7 | .423 | .989 |
| 7.5 | .385 | .99 |
| 8 | .330 | .992 |
| 8.5 | .302 | .993 |
| 9 | .278 | .995 |
| 9.5 | .258 | .995 |
| 10 | .241 | .996 |
| 10.5 | .220 | .997 |
| 11 | .186 | .998 |
| 11.5 | .155 | .998 |
| 12 | .131 | .998 |
| 12.5 | .103 | .999 |
| 13 | .086 | .999 |
| 13.5 | .052 | .999 |
| 14 | .048 | .999 |
| 14.5 | .027 | 1 |
| 15 | .017 | 1 |
| 15.5 | .014 | 1 |
| 16 | .010 | 1 |
| 16.5 | .003 | 1 |
| 17* | . | . |
| 17.5* | . | . |
| 18.0 | .000 | 1 |

* Sensitivity and specificity could not be calculated as no individuals had this A-TAC score

**Disorder: ADHD (Total)**

| **A-TAC score** | **Sensitivity** | **Specificity** |
| --- | --- | --- |
| 0 | 1.0 | .0 |
| .5 | .936 | .412 |
| 1 | .911 | .523 |
| 1.5 | .890 | .608 |
| 2 | .876 | .67 |
| 2.5 | .842 | .721 |
| 3 | .806 | .763 |
| 3.5 | .773 | .799 |
| 4 | .753 | .829 |
| 4.5 | .722 | .854 |
| 5 | .685 | .875 |
| 5.5 | .668 | .895 |
| 6 | .636 | .91 |
| 6.5 | .612 | .922 |
| 7 | .575 | .933 |
| 7.5 | .533 | .943 |
| 8 | .506 | .951 |
| 8.5 | .471 | .958 |
| 9 | .448 | .963 |
| 9.5 | .417 | .968 |
| 10 | .384 | .972 |
| 10.5 | .361 | .977 |
| 11 | .336 | .98 |
| 11.5 | .324 | .983 |
| 12 | .297 | .985 |
| 12.5 | .278 | .987 |
| 13 | .252 | .989 |
| 13.5 | .226 | .991 |
| 14 | .208 | .992 |
| 14.5 | .191 | .994 |
| 15 | .167 | .995 |
| 15.5 | .138 | .996 |
| 16 | .119 | .997 |
| 16.5 | .096 | .998 |
| 17 | .088 | .998 |
| 17.5 | .067 | .999 |
| 18 | .043 | .999 |
| 18.5 | .031 | .999 |
| 19 | .02 | 1 |
| 19.5* | . | . |
| 20.0 | .00 | .00 |

* Sensitivity and specificity is not presented since no subject had this A-TAC score.
