# Supplementary material for: The Autism–Tics, ADHD and other Comorbidities inventory (A-TAC): previous and predictive validity
Source: BMC Psychiatry. 2017 Dec 16;17:403. doi: 10.1186/s12888-017-1563-0 (PMC5732476; doi:10.1186/s12888-017-1563-0)
Supplement: Supplementary file 3 — CATSS data stratified by age. Table S1. and Table S2. include data from the 12- year-olds, born 1st of July 1992 to 30th of June 1995. Table S3. and Table S4. include data from the 9-year-olds, born 1st of July 1995 and onwards. (DOCX 31 kb) [file 12888_2017_1563_MOESM3_ESM.docx]

**CATSS data stratified by age**

Table 1 and Table 2 include data from the 12- year-olds, born 1^st^ of July 1992 to 30^th^ of June 1995. Table 3 and Table 4 include data from the 9-year-olds, born 1^st^ of July 1995 and onwards.

**12-year-olds**

Table 1

*Subjects With a Listed Disorder in NPR (%) and Screen-positives for Each Cut-off Value (%)*

|  | Disorder in NPR | | |  | |
| --- | --- | --- | --- | --- | --- |
| Disorder | Before | After | Total | Cut-off | Screen-positive |
| ASD | 29 (0.4) | 65 (1.0) | 94 (1.4) | 4.5 | 245 (3.8) |
|  |  |  |  | 8.5 | 67 (1.0) |
| ADHD | 57 (0.9) | 165 (2.5) | 222 (3.4) | 6 | 649 (10.0) |
|  |  |  |  | 12.5 | 118 (1.8) |
| LD | 43 (0.7) | 36 (0.6) | 79 (1.2) | 1 | 1128 (17.3) |
|  |  |  |  | 3 | 128 (2.0) |
| DCD | 13 (0.2) | 6 (0.1) | 19 (0.3) | 0.5 | 552 (8.5) |
|  |  |  |  | 1 | 136 (2.1) |
| TD | 18 (0.3) | 14 (0.2) | 32 (0.5) | 1.5 | 220 (3.4) |
| ODD | 3 (0.0) | 7 (0.1) | 10 (0.2) | 3 | 188 (2.9) |
| CD | 7 (0.1) | 23 (0.4) | 30 (0.5) | 2 | 54 (0.8) |
| OCD^a^ | 3 (0.0) | 30 (0.5) | 33 (0.5) | 1 | 154 (2.4) |
| ED^a^ | 21 (0.3) | 68 (1.0) | 89 (1.4) | 1 | 389 (6.0) |

*N* = 6506.

Disorder in NPR: Ascribed diagnoses in NPR before and after the telephone interview (CATSS) and in total.

A-TAC: Number of screen-positive subjects with an A-TAC score equal to or higher than each cut-off value.

^a^ No previous established cut-off values.

Table 2

*Previous and Predictive Validity of A-TAC.*

|  |  | Previous | | Predictive | | Total | |
| --- | --- | --- | --- | --- | --- | --- | --- |
| Disorder | Cut-off | AUC | sens/spec | AUC | sens/spec | AUC | sens/spec |
| ASD | 4.5 (low) | 0.982 | 0.862/0.966 | 0.793 | 0.453/0.966 | 0.854 | 0.581/0.97 |
|  | 8.5 (high) |  | 0.483/0.992 |  | 0.172/0.991 |  | 0.269/0.993 |
| ADHD | 6 (low) | 0.912 | 0.754/0.906 | 0.835 | 0.612/0.913 | 0.860 | 0.649/0.919 |
|  | 12.5 (high) |  | 0.439/0.986 |  | 0.164/0.986 |  | 0.234/0.989 |
| LD | 1 (low) | 0.867 | 0.814/0.831 | 0.898 | 0.861/0.83 | 0.883 | 0.835/0.835 |
|  | 3 (high) |  | 0.372/0.983 |  | 0.417/0.983 |  | 0.392/0.985 |
| DCD | 0.5 (low) | 0.843 | 0.750/0.916 | 0.546 | 0.167/0.915 | 0.744 | 0.556/0.916 |
|  | 1 (high) |  | 0.417/0.98 |  | 0.167/0.979 |  | 0.333/0.98 |
| TD | 1.5 | 0.865 | 0.500/0.967 | 0.669 | 0.214/0.967 | 0.780 | 0.375/0.968 |
| ODD | 3 | 0.992 | 1.0/0.972 | 0.938 | 0.571/0.972 | 0.955 | 0.700/0.972 |
| CD | 2 | 0.832 | 0.571/0.992 | 0.700 | 0.174/0.992 | 0.731 | 0.267/0.993 |
| OCD^a^ | 1 | 0.992 | 1.0/0.977 | 0.696 | 0.300/0.978 | 0.723 | 0.364/0.978 |
| ED^a^ | 1 | 0.750 | 0.381/0.941 | 0.522 | 0.118/0.941 | 0.576 | 0.180/0.942 |

*N* = 6506.

Previous and predictive validity: area under the receiver operating characteristics curve (AUC) and sensitivity/specificity for each cut-off value in the A-TAC.

^a^ No previous established cut-off value.

**9-year-olds**

Table 3

*Subjects With a Listed Disorder in NPR (%) and Screen-positives for Each Cut-off Value (%)*

|  | Disorder in NPR | | |  | |
| --- | --- | --- | --- | --- | --- |
| Disorder | Before | After | Total | Cut-off | Screen-positive |
| ASD | 98 (0.5) | 106 (0.5) | 204 (1.1) | 4.5 | 679 (3.5) |
|  |  |  |  | 8.5 | 199 (1.0) |
| ADHD | 174 (0.9) | 327 (1.7) | 501 (2.6) | 6 | 2058 (10.7) |
|  |  |  |  | 12.5 | 406 (2.1) |
| LD | 102 (0.5) | 68 (0.4) | 170 (0.9) | 1 | 2833 (14.7) |
|  |  |  |  | 3 | 300 (1.6) |
| DCD | 57 (0.3) | 12 (0.1) | 69 (0.4) | 0.5 | 1498 (7.8) |
|  |  |  |  | 1 | 333 (1.7) |
| TD | 23 (0.1) | 33 (0.2) | 56 (0.3) | 1.5 | 627 (3.2) |
| ODD | 5 (0.0) | 13 (0.1) | 18 (0.1) | 3 | 607 (3.1) |
| CD | 20 (0.1) | 27 (0.1) | 47 (0.2) | 2 | 224 (1.2) |
| OCD^a^ | 6 (0.0) | 31 (0.2) | 37 (0.2) | 1 | 325 (1.7) |
| ED^a^ | 11 (0.1) | 59 (0.3) | 70 (0.4) | 1 | 982 (5.1) |

*N* = 19322.

Disorder in NPR: Ascribed diagnoses in NPR before and after the telephone interview (CATSS) and in total.

A-TAC: Number of screen-positive subjects with an A-TAC score equal to or higher than each cut-off value.

^a^ No previous established cut-off values.

Table 4

*Previous and Predictive Validity of A-TAC.*

|  |  | Previous | | Predictive | | Total | |
| --- | --- | --- | --- | --- | --- | --- | --- |
| Disorder | Cut-off | AUC | sens/spec | AUC | sens/spec | AUC | sens/spec |
| ASD | 4.5 (low) | 0.983 | 0.840/0.969 | 0.824 | 0.394/0.967 | 0.902 | 0.606/0.971 |
|  | 8.5 (high) |  | 0.479/0.992 |  | 0.173/0.991 |  | 0.318/0.993 |
| ADHD | 6 (low) | 0.935 | 0.801/0.899 | 0.814 | 0.540/0.901 | 0.860 | 0.630/0.907 |
|  | 12.5 (high) |  | 0.462/0.983 |  | 0.210/0.982 |  | 0.297/0.986 |
| LD | 1 (low) | 0.873 | 0.833/0.857 | 0.932 | 0.912/0.856 | 0.898 | 0.865/0.86 |
|  | 3 (high) |  | 0.373/0.986 |  | 0.397/0.986 |  | 0.382/0.988 |
| DCD | 0.5 (low) | 0.813 | 0.684/0.924 | 0.587 | 0.250/0.923 | 0.774 | 0.609/0.924 |
|  | 1 (high) |  | 0.368/0.984 |  | 0.083/0.983 |  | 0.319/0.984 |
| TD | 1.5 | 0.856 | 0.652/0.968 | 0.849 | 0.576/0.968 | 0.853 | 0.607/0.969 |
| ODD | 3 | 0.989 | 0.800/0.969 | 0.722 | 0.385/0.969 | 0.796 | 0.500/0.969 |
| CD | 2 | 0.928 | 0.500/0.989 | 0.696 | 0.222/0.989 | 0.795 | 0.340/0.989 |
| OCD^a^ | 1 | 0.819 | 0.500/0.983 | 0.610 | 0.194/0.983 | 0.644 | 0.243/0.984 |
| ED^a^ | 1 | 0.638 | 0.364/0.949 | 0.504 | 0.085/0.949 | 0.525 | 0.129/0.949 |

*N* = 19 322

Previous and predictive validity: area under the receiver operating characteristics curve (AUC) and sensitivity/specificity for each cut-off value in the A-TAC.

^a^ No previous established cut-off value.
